# A predictive nomogram: a cross-sectional study on a simple-to-use model for screening 12-year-old children for severe caries in middle schools

**DOI:** 10.1186/s12903-021-01819-2

**Published:** 2021-09-20

**Authors:** Shaoying Duan, Meng Li, Jialiang Zhao, Huiyu Yang, Jinfeng He, Lei Lei, Ran Cheng, Tao Hu

**Affiliations:** 1 Department of Preventive Dentistry, State Key Laboratory of Oral Diseases, National Clinical Research Center for Oral Diseases, West China Hospital of Stomatology, Sichuan University, Chengdu, Sichuan China; 2 Orange Dental Technology Co., Ltd., Shanghai, China

**Keywords:** Epidemiology, Caries risk, Oral health, Regression analysis, Models, Statistical, Cross-sectional studies

## Abstract

**Background:**

A nomogram is a tool that transforms complex regression equations into simple and visual graphs and enables clinicians and patients to conveniently compute output probabilities without needing medical knowledge and complex formulas. The aim of this study was to develop and validate a predictive nomogram to screen for severe caries among 12-year-old children based on risk factors in Sichuan Province, China.

**Methods:**

A cross-sectional study of 4573 12-year-olds was conducted up to May 2016 in middle schools from three districts and three counties in Sichuan Province, China. All the children underwent oral examinations and completed questionnaires to assess general information, oral impacts on daily performance, dietary habits, subjective health conditions, history of dental trauma, frequency of toothache, dental visits, and knowledge, attitudes, and behaviours toward oral hygiene. Univariate analysis and multivariate logistic regression analysis were used to determine which variables were significantly associated with severe caries (operationalized as DMFT ≥ 3). A nomogram was developed and validated by using the ‘rms’ package and two cross-validation methods.

**Results:**

Severe caries was found in 537 of the 4573 children (11.74%). Multivariate logistic regression analysis revealed that the following variables predicted a higher risk of severe caries: ‘female’ [odds ratio (OR) = 1.985, 95% confidence interval (95% CI): 1.63–2.411], ‘urban’ (OR = 2.389, 95% CI: 1.96–2.91), ‘non-only child’ (OR = 1.317, 95% CI: 1.07–1.625), ‘very poor self-assessment of oral health status’ (OR = 2.157, 95% CI: 1.34–3.467) and ‘visited a dentist less than 6 months’ (OR = 1.861, 95% CI: 1.38–2.505). Multivariate logistic regression analysis also indicated that the following variables predicted a lower risk of severe caries: ‘middle level of urbanization’ (OR = 0.395, 95% CI: 0.32–0.495) and ‘high level of urbanization’ (OR = 0.466, 95% CI: 0.37–0.596). Both the fivefold and leave-one-out cross-validation methods indicated that the nomogram model built by these 6 variables displayed good disease recognition ability.

**Conclusions:**

The nomogram was a simple-to-use model to screen children for severe caries. This model was found to facilitate non-dental professionals in assessing risk values without oral examinations and making referrals to dental professionals.

**Supplementary Information:**

The online version contains supplementary material available at 10.1186/s12903-021-01819-2.

## Background

Dental caries is one of the most prevalent chronic diseases; it occurs among susceptible children who are at risk for developing decay and progresses throughout their life spans [1, 2]. Dental caries also diminishes oral health-related quality of life [3]. The global oral health goal was that by the year 2000, the mean decayed, missing, and filled teeth (DMFT) index among 12-year-old children would be no more than 3, which was accepted for caries prevention by the World Health Organization (WHO) and the International Dental Federation (FDI) in 1981 [4, 5]. Although China has maintained a mean DMFT of ≤ 1.00 since 1983 owing to the use of fluoride and the achievement of oral health education [6], the distribution of caries remains skewed. It has been reported that most cases of caries are found in only a small number of children [7, 8]. Thus, we can obtain the Significant Caries (SiC) Index of a certain area by examining one-third of the local children who have the highest number of DMFT. According to the global goal of caries levels that was proposed in 2000, the SiC index should have been less than 3 DMFT among 12-year-old children by 2015 [9]. Screening out children with severe caries and taking targeted preventive measures will help save socioeconomic resources, improve caries-related outcomes and contribute to better oral health.

Data from the World Health Survey showed that oral healthcare coverage was 46.5% in China [10]. Located in southwestern China, Sichuan Province had a population of 83.41 million in 2018, but there were only 9,225 dental practitioners and assistants [11]. Moreover, Chinese parents rarely take their children to regular oral examinations. Therefore, a simple-to-use predictive model is required to help non-dental professionals (e.g., medical staff at community facilities or school doctors) assess risk values without oral examinations and make referrals to dental professionals.

Nomograms are widely used as reliable risk prediction tools [12–14] and can generate a numeral probability by integrating risk variables. This model is simple and rapid for application because it does not require complex mathematical formulas or medical knowledge. Although there have been studies on nomograms in the dental field [15], to the best of our knowledge, few studies have formulated nomograms suitable for screening for severe caries.

This article aimed to investigate risk factors for severe caries in children and to develop and validate a simple-to-use nomogram to screen for severe caries among 12-year-old children based on the risk level calculated in Sichuan Province, China.

## Methods

A cross-sectional survey of children aged 12 years was conducted in Sichuan Province between December 2015 and May 2016. Ethics approval of the Oral Health Survey was obtained from the Stomatological Ethics Committee of the Chinese Stomatological Association and the Ethics Committee of the West China Hospital of Stomatology, Sichuan University (Approval No. 2014-003). Consent for participation and publication was also obtained from the parents or legal guardians of the children in this study.

### Sampling

A multistage stratified random-cluster survey sampling design was used for participant selection [16]. In the first stage, based on the 2010 census conducted by the National Bureau of Statistics of the People’s Republic of China, three districts and three counties in Sichuan Province (Guang’an, Chuanshan, and Jinniu Districts, Yibin, Da, and Pi Counties) were randomly selected to represent low/middle/high levels of urbanization by probability-proportional-to-size (PPS) [17]. Next, a simple random sampling method was used to select middle schools. Consisting of 3 private schools and 28 public schools, a total number of 31 randomly-selected schools got involved in the study. Finally, all 12-year-old children from the selected schools in each area were invited to participate in the study. The ages were calculated according to the survey month. The sample size was calculated based on the following formula:

$$n = deff\frac{{\mu^{2} (1 - p)}}{{\varepsilon^{2} p(1 - nonresponse)}},$$

where n is the sample size, the design effect *deff* is 2.5, *p* is 28.9% according to the dental caries prevalence in the Third National Oral Health Survey, *μ* (1.96) is the level of confidence, *ε* (7.5%) is the margin of error, and the nonresponse rate is 5% [18]. The formula above indicated that a sample size of 4420 12-year-old children was required.

### Quality control

To ensure inter-examiner reliability, 4 trained and licensed dentists, including three training examiners and one calibrating examiner, were required to receive the pre-survey calibration training. The results of caries examination for schoolchildren were recorded by assistants. To ensure intra-examiner reliability of oral examination, 5% of samples were selected for duplicate examination, then compared with the original data and with the calibrating examiner every day during the survey. Cohen’s kappa statistics assessing the consistency of inter- and intra-examiner were all exceeded 0.80 [19].

### Caries examination

After parents signed informed consent forms, all 4800 schoolchildren who participated in questionnaire surveys were examined on mobile dental chairs with portable lights at the schools. A tooth was classified as decayed when there was a lesion in a pit or fissure; on a smooth tooth surface with an unmistakable cavity; on undermined enamel; or on a detectably softened floor or wall. The DMFT of permanent teeth examinations mainly relied on ocular inspection with the help of Community Periodontal Index (CPI) probes as recommended by the WHO for clinical examinations [20]. This DMFT index covers the teeth and/or tooth number that were decayed, filled or extracted as a result of caries, which we used to assess dental caries.

### Questionnaire

Thirty-nine closed questions (see Additional file 1) were designed by experts based on the variables suggested by the WHO [20]. Paper printed questionnaires were completed by children individually in the classroom under the explanations of one licenced dentist. If a child did not fill the questionnaires completely or did not qualify, they were excluded from the analyses.

### Independent variables

Thirty-nine independent variables came from questionnaire containing children’s socio-demographic information, including age, gender, districts/counties (Guang’an, Chuanshan, and Jinniu Districts, Yibin, Da, and Pi Counties) represent different level of urbanization, region (urban/rural), father/mother’s education level (illiterate/low/medium/high), only child (yes/no) and oral impacts on daily performances (serious/general/slight/none/does not know; impact on eating, talking, brushing, working, schooling, sleeping, grinning, communicating, and if easily troubled). Additionally, questions assessing dietary habits, subjective health conditions, history of dental trauma, frequency of toothache in the previous 12 months, dental visits, and knowledge, attitudes, and behaviours toward oral hygiene were also included.

### Outcome variable selection

The global goal for dental caries prevention among 12-year-olds was an SiC Index less than 3 DMFT [9]. Therefore, the outcome variable in this study was set as a binary variable based on whether a child had severe caries (DMFT ≥ 3).

### Statistical analysis

Statistical analyses were performed using SAS 9.4 (SAS Institute, Inc., Cary, NC) for Windows. Univariate analyses (chi-squared tests) were first conducted to locate factors that might be associated with the outcome variable. Second, a multivariate logistic regression model was built to evaluate the association of the outcome variables with the selected variables mentioned above using a backward selection method (entry significance level = 0.05, stay significance level = 0.10) [21, 22]. The partial regression coefficient (β), Wald’s χ^2^, *P*-value (Wald test), odds ratio (OR) and 95% confidence intervals (95% CI) were estimated to specify the predictive model. Third, a nomogram was constructed based on the results of a multivariate logistic regression model by using the ‘rms’ package in R version 2.14.1 (http://www.r-project.org/). Finally, the prediction performance of the model was assessed by using both fivefold and leave-one-out cross-validation (CV) methods. The fivefold CV method was conducted according to the following steps: (i) data were randomly divided into five parts; (ii) one-fifth of the data was set aside as a validation dataset, and a logistic regression model was fitted using the remaining four-fifths (the training dataset); (iii) the resulting training model was used to calculate the predicted probability of each validation observation; (iv) steps (i) to (iii) were repeated four more times; and (v) average indicators were calculated. Indicators, including sensitivity, specificity, false-positive rate (FPR), false-negative rate (FNR), negative predictive value (NPV), positive predictive value (PPV), Youden Index (YI), accuracy, and area under the curve (AUC), were calculated for training, validation, and all data to assess the predictive ability of the model. For a similar reason, the leave-one-out CV method was used to fit the model for all cases except one and then tested on the set-aside case. This process was repeated for each observation in the original sample (random sampling without replacement). A receiver operating characteristic (ROC) curve and a calibration curve were also generated to assess the predictive ability of the model. All statistical tests (except stay significance level of multivariate logistic regression model) were two-tailed with the significance levels set to 0.05.

## Results

### Socio-demographic characteristics

A total of 4800 children participated in the survey. Among them, 227 children were excluded due to failure to complete questionnaires. Ultimately, 4573 children were included in this study, and the response rate was 94.27%. DMFT index and its components were presented in Table 1. Among the 4573 12-year-olds, 2248 (49.16%) had experience with caries (DMFT ≥ 1), including 1048 boys and 1200 girls. Only 537 (11.74%) children had severe caries (DMFT ≥ 3), which revealed a significantly skewed distribution.

**Table 1:** Dental caries status of 12-year-old children in Sichuan province, 2015–2016

Variable	n (%)	DMFT	D		M		F	
		Mean (SD)	Mean (SD)	Rate (%)	Mean (SD)	Rate (%)	Mean (SD)	Rate (%)
*Gender*								
Male	2227 (48.7)	0.64 (1.29)	0.59 (1.22)	29.23	0.00 (0.02)	0.04	0.05 (0.34)	3.05
Female	2346 (51.3)	1.07 (1.73)	0.95 (1.62)	40.41	0.00 (0.05)	0.09	0.11 (0.54)	5.80
*Region*								
Urban	2275 (49.7)	1.05 (1.68)	0.98 (1.63)	41.30	0.00 (0.02)	0.04	0.07 (0.39)	3.57
Rural	2298 (50.3)	0.67 (1.36)	0.57 (1.21)	28.57	0.00 (0.05)	0.09	0.10 (0.51)	5.36
*Urbanization*								
Low	1614 (35.3)	1.13 (1.77)	1.10 (1.73)	43.68	0.00 (0.03)	0.06	0.04 (0.29)	1.98
Middle	1680 (36.7)	0.68 (1.37)	0.60 (1.26)	30.00	0.00 (0.02)	0.06	0.07 (0.45)	4.05
High	1279 (28.0)	0.75 (1.40)	0.59 (1.12)	30.49	0.00 (0.06)	0.08	0.15 (0.60)	8.13
*Mother’s education level*								
Illiterate	832 (18.2)	0.88 (1.64)	0.80 (1.56)	34.98	0.00 (0.03)	0.12	0.08 (0.43)	4.57
Low	905 (19.8)	0.82 (1.44)	0.76 (1.37)	35.03	0.00 (0.03)	0.11	0.06 (0.34)	3.31
Medium	1677 (36.7)	0.89 (1.57)	0.82 (1.50)	36.85	0.00 (0.00)	0.00	0.07 (0.42)	3.58
High	1159 (25.3)	0.83 (1.50)	0.70 (1.35)	32.18	0.00 (0.06)	0.09	0.13 (0.58)	6.56
*Father's education level*								
Illiterate	775 (16.9)	0.89 (1.61)	0.80 (1.51)	36.00	0.00 (0.05)	0.26	0.09 (0.45)	5.03
Low	678 (14.8)	0.72 (1.32)	0.67 (1.24)	34.07	0.00 (0.08)	0.15	0.05 (0.36)	2.36
Medium	1704 (37.3)	0.89 (1.62)	0.84 (1.56)	36.09	0.00 (0.00)	0.00	0.05 (0.35)	3.11
High	1416 (31.0)	0.86 (1.51)	0.73 (1.37)	33.47	0.00 (0.00)	0.00	0.13 (0.59)	6.78
*Only child*								
Yes	1665 (36.4)	0.75 (1.43)	0.62 (1.26)	31.05	0.00 (0.05)	0.06	0.13 (0.59)	6.37
No	2908 (63.6)	0.92 (1.60)	0.86 (1.54)	37.21	0.00 (0.03)	0.07	0.06 (0.35)	3.37
Total	4573 (100.0)	0.86 (1.54)	0.77 (1.45)	34.97	0.00 (0.00)	0.07	0.08 (0.45)	4.46

### Univariate analysis

Univariate analyses (chi-squared tests) were conducted first, and 16 factors were found to be statistically significantly associated with DMFT ≥ 3 (*P* < 0.05; Table 2): socio-demographic information (‘gender’, ‘region’, ‘only child’, ‘urbanization’), cariogenic diet (‘candy/chocolate/cookies/cakes’), self-assessment (‘self-assessment of oral health status’), trauma (‘history of dental trauma’), toothache (‘frequency of toothache in the previous 12 months’), dental visit (‘history of dental visits’, ‘reason for last dental visit’, ‘time of last dental visit’), oral hygiene knowledge and attitude (‘fluoride can protect teeth’, ‘pit and fissure sealing can protect teeth’, ‘regular dental check is necessary’), and oral impacts on daily performances (‘impact on sleeping’, ‘impact on grinning’).

**Table 2:** Description of the potential risk indicators selected by univariate analysis

Variables	DMFT ≥ 3 (%)	DMFT < 3 (%)	χ^2^	*P*
*Gender*				
Male	183 (8.22)	2044 (91.78)	52.0611	< 0.0001***
Female	354 (15.09)	1992 (84.91)		
*Region*				
Urban	361 (15.87)	1914 (84.13)	74.0525	< 0.0001***
Rural	176 (7.66)	2122 (92.34)		
*Urbanization*				
Low	280 (17.35)	1334 (82.65)	77.6834	< 0.0001***
Middle	134 (7.98)	1546 (92.02)		
High	123 (9.61)	1156 (90.38)		
*Candy/chocolate/cookies/cakes*				
Never/seldom	46 (8.80)	477 (91.20)	14.3734	0.0134**
1–3 times/month	57 (9.61)	536 (90.39)		
Once/week	75 (10.55)	636 (89.45)		
2–6 times/week	167 (12.46)	1173 (87.54)		
Once/day	107 (13.02)	715 (86.98)		
≥ 2 times/day	85 (14.55)	499 (85.45)		
*Only child*				
Yes	161 (9.67)	1504 (90.33)	10.8584	0.0010**
No	376 (12.93)	2532 (87.07)		
*Self-assessment of oral health status*				
Very good	8 (6.25)	120 (93.75)	30.5292	< 0.0001***
Good	98 (9.82)	900 (90.18)		
Average	288 (11.14)	2298 (88.86)		
Poor	117 (15.88)	620 (84.12)		
Very poor	26 (20.97)	98 (79.03)		
*History of dental trauma*				
Yes	141 (11.80)	1054 (88.20)	6.0979	0.0474*
No	212 (10.59)	1790 (89.41)		
Don’t remember	184 (13.37)	1192 (86.63)		
*Frequency of toothache in the previous 12 months*				
Often	20 (18.18)	90 (81.82)	11.6055	0.0089**
Occasionally	320 (12.50)	2241 (87.50)		
Never	129 (9.63)	1210 (90.37)		
Don’t remember	68 (12.08)	495 (87.92)		
*History of dental visits*				
Yes	286 (13.41)	1847 (86.59)	10.6997	0.0011**
No	251 (10.29)	2189 (89.71)		
*Time of last dental visit*				
Never	251 (10.29)	2191 (89.71)	16.8472	0.0008***
< 6 months ago	76 (16.70)	379 (83.30)		
6–12 months ago	65 (12.17)	469 (87.83)		
> 12 months ago	145 (12.68)	999 (87.33)		
*Reason for last dental visit*				
Never/don’t know	408 (11.15)	3251 (88.85)	15.2422	0.0016 **
Consultation	34 (12.98)	228 (87.02)		
Prevention	6 (5.94)	95 (94.06)		
Treatment	89 (16.15)	462 (83.85)		
*Fluoride can protect teeth*				
Correct	371 (11.15)	2957 (88.85)	4.1757	0.0410*
Wrong/don’t know	166 (13.33)	1079 (86.67)		
*Pit and fissure sealing can protect teeth*				
Correct	460 (11.39)	3578 (88.61)	4.1044	0.0428*
Wrong/don’t know	77 (14.39)	458 (85.61)		
*Regular dental check is necessary*				
Agree	381 (12.25)	2730 (87.75)	7.8628	0.0489*
Disagree	8 (6.67)	112 (93.33)		
Perhaps	81 (9.75)	750 (90.25)		
Don’t know	67 (13.11)	444 (86.89)		
*Impact on sleeping*				
Serious	18 (14.75)	104 (85.25)	21.6344	0.0002***
General	45 (17.72)	209 (82.28)		
Slight	100 (13.81)	624 (86.19)		
None	357 (11.15)	2846 (88.85)		
Don’t know	17 (6.30)	253 (93.70)		
*Impact on grinning*				
Serious	38 (13.67)	240 (86.33)	11.9381	0.0178*
General	60 (11.28)	472 (88.72)		
Slight	142 (13.63)	900 (86.37)		
None	278 (11.41)	2159 (88.59)		
Don’t know	19 (6.69)	265 (93.31)		

^*^P < 0.05, ** *P* < 0.01, *** *P* < 0.001

### Multivariate logistic regression

A multivariate logistic regression model was built with the selected variables. For the predictive model, the following variables were included: ‘female’, ‘urban’, ‘low level of urbanization’, ‘only child’, ‘poor or very poor oral health for self-assessment’, and ‘visited a dentist less than 6 months or more than 12 months ago’ (*P* < 0.1; Table 3).

**Table 3:** Selected variables associated with the risk of DMFT ≥ 3 in the final regression model

Independent variables	*β*	Wald χ^2^	*P*	OR	95% CI	
					Lower	Upper
*Intercept*	− 3.8054	189.997	< 0.0001***	–	–	–
*Region (vs. Rural)*						
Urban	0.871	74.8896	< 0.0001***	2.389	1.96	2.91
*Urbanization (vs. Low)*						
Middle	− 0.9299	65.1115	< 0.0001***	0.395	0.32	0.495
High	− 0.7631	36.9668	< 0.0001***	0.466	0.37	0.596
*Gender (vs. Male)*						
Female	0.6854	47.5841	< 0.0001***	1.985	1.63	2.411
*Only child (vs. Yes)*						
No	0.2752	6.5702	0.0104*	1.317	1.07	1.625
*Self-assessment of oral health status (vs. Average)*						
Very good	− 0.657	3.0272	0.0819	0.518	0.25	1.087
Good	− 0.1532	1.427	0.2322	0.858	0.67	1.103
Poor	0.4256	11.8617	0.0006***	1.53	1.2	1.95
Very poor	0.7687	10.0731	0.0015**	2.157	1.34	3.467
*Time of last dental visit (vs. Never)*						
< 6 months ago	0.6213	16.8055	< 0.0001***	1.861	1.38	2.505
6–12 months ago	0.1848	1.4227	0.233	1.203	0.89	1.63
> 12 months ago	0.3287	7.7635	0.0053**	1.389	1.1	1.75

^*^P < 0.05, ** *P* < 0.01, *** *P* < 0.001

### Nomogram formulation

**Fig. 1:**
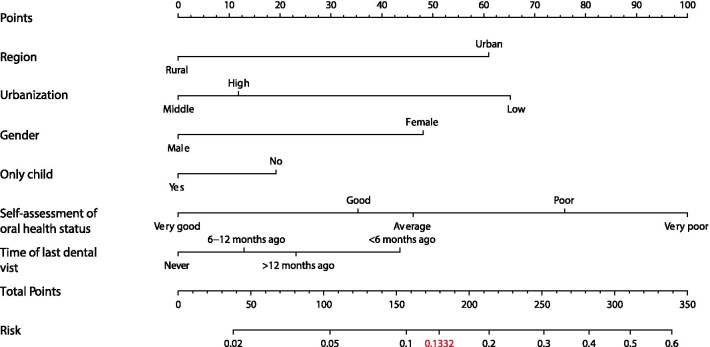
The nomogram constructed for identifying severe caries was based on multivariate logistic regression. This model is quite simple to use. For example, if a child is in an urban population, a vertical line is drawn where the region of the nomogram is “urban”, and the corresponding points are approximately 60 points. Similarly, suppose this child satisfies the conditions of “Female”, “Middle level of urbanization”, “Non-only child”, “Very poor oral health for self-assessment” and “never visits dentist”, and the scores are “48, 0, 20, 100, 0” respectively. In this case, the total points are 228, and the corresponding risk value is between 0.2 and 0.3

A simple-to-use nomogram was formulated based on six risk factors for the children with severe caries by multivariate logistic regression (Fig. 1). The longer the variable scales were, the more relative importance they had. ‘Risk’ indicated the possibility of a child with severe caries (DMFT ≥ 3), and the cut-off point was 0.1332 based on the ROC curve.

### Model validation

**Fig. 2:**
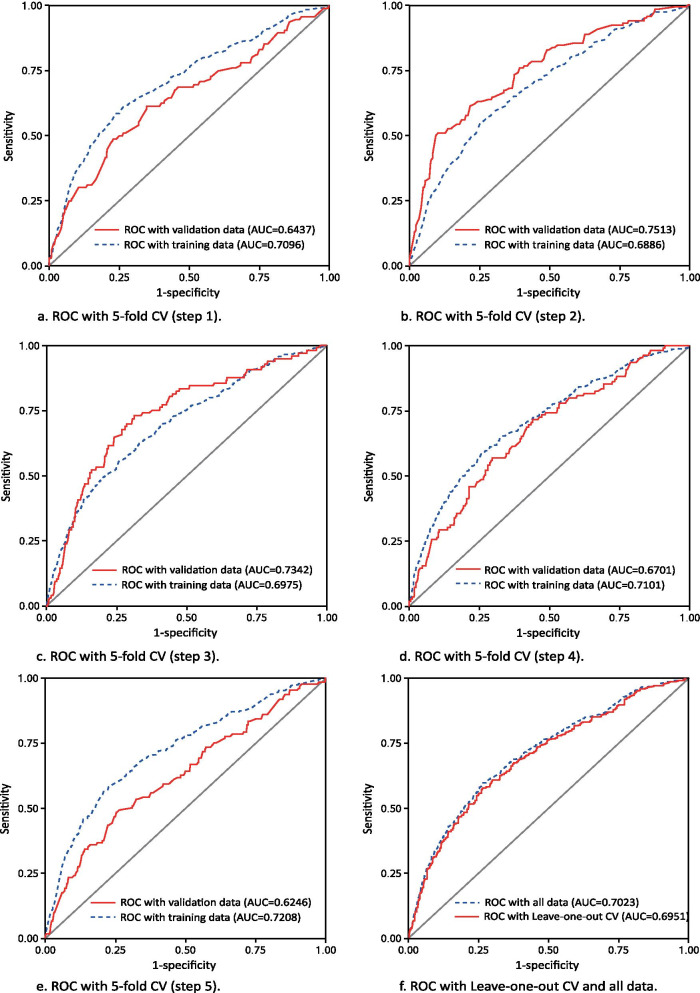
Nomogram ROC curves with fivefold CV and leave-one-out CV. Five ROC curves (**a**–**e**) with validation data (red solid line) and training data (blue dashed line) and one ROC curve (**f**) with leave-one-out CV (red solid line) and all data (blue dashed line)

**Fig. 3:**
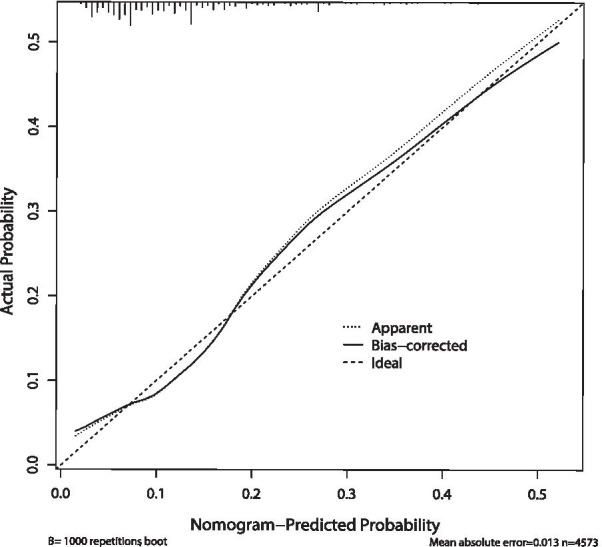
The calibration curve of the nomogram model. For the calibration curve, the X-axis is the predicted probability, and the Y-axis is the actual probability. The diagonal (ideal curve/dashed line) meant the prediction probability was completely consistent with the actual rate, and our curve (apparent curve/solid line) was close to the diagonal, which suggested that this model shows good predictive performance

We assessed the discrimination performance of the model using fivefold CV and leave-one-out CV methods, providing five ROC curves with both validation data and training data (Fig. 2). The mean areas under the ROC curve (AUCs) were 0.6848 (95% CI: 0.6157, 0.7539) for the training data and 0.7053 (95% CI: 0.6898, 0.7208) for the validation data with fivefold CV. The AUCs were 0.7023 (95% CI: 0.67682, 0.72778) (Table 4) for all data and 0.6951 for leave-one-out CV (Fig. 2). Additionally, a calibration curve was made to assess the predictive performance of the nomogram (Fig. 3). Sensitivity, specificity, YI, accuracy, FPR, FNR, NPV and PPV were also calculated (Table 4).

**Table 4:** Validation and evaluation indicators of the screening model in training and validation data

Indicators	Training data	Validation data
Sensitivity	66.45 (64.33, 68.57)	66.03 (64.30, 67.77)
Specificity	64.26 (59.61, 68.91)	63.88 (61.56, 66.20)
False positive rate (FPR)	35.74 (31.09, 40.39)	36.12 (33.80, 38.44)
False negative rate (FNR)	33.55 (31.43, 35.67)	33.97 (32.23, 35.70)
Positive predictive value (PPV)	19.90 (18.43, 21.38)	19.57 (18.94, 20.20)
Negative predictive value (NPV)	93.50 (93.16, 93.85)	93.40 ((93.26, 93.54)
Youden Index (YI)	30.71 (27.36, 34.06)	29.91 (28.96, 30.86)
Accuracy (ACC)	64.53 (60.61, 68.44)	64.13 (62.27, 65.99)
Area under curve (AUC)	70.53 (68.98,72.08)	68.48 (61.57, 75.39)

## Discussion

The global prevalence and severity of caries are different between developing and developed countries [23, 24], but the first challenge we face is the same: screening out people with severe caries and taking targeted preventive measures [25]. Many caries risk assessment (CRA) tools have been built in previous research on risk factor management, including high-cost examinations such as salivary flow and composition, cariogenic bacteria, and genetic factors [26]. Although these methods improve the accuracy of the predictive model, they require more waiting time, qualified dentists, laboratory technicians, and funds. There is a shortage of dental professionals in China, and the number of children suffering from tooth decay is large. For this reason, these methods are not applicable for caries screening in large-scale populations.

In our study, a simple-to-use predictive model was conducted based on dental examinations and questionnaires from a cross-sectional survey in Sichuan Province. The cumulative points of every independent variable in the nomogram could be calculated and matched to the scale of risk possibility. We recommend a two-stage stratified screening for severe caries among 12-year-old children in Sichuan Province: (i) The proposed nomogram is used in the first stage to reduce manpower and financial resources. Non-dental professionals administer questionnaires involving six risk factors and calculate the risk value of severe caries according to the model. If a risk value is higher than 0.1332 (cut-off point), it is recommended to visit a dentist as early as possible and enter the second stage; if the value is below the cut-off point, regular oral examinations (two times per year) are recommended. (ii) In the second stage, CRA tools or other oral examinations are conducted by dental specialists to further assess the severity of caries.

Risk factors in our study were mainly associated with socio-demographic information and dental experiences. In accordance with previous studies [27, 28], “female children” have a higher risk factor for caries of permanent teeth. Their caries prevalence is associated with earlier tooth eruption [29], low salivary flow rate, sugar consumption [30] and dental phobia [31, 32], which may require improvements in dental education, medical environmental design, and doctor-patient communication.

Caries experience was the primary risk factor [33, 34]. The relationship between caries experience and dental visit information was corroborated by previous research [35, 36]. It seems contradictory that children who visit the dentist more often have a higher likelihood of caries. A possible reason for this is that many parents take their children to the dentist only for treatment instead of prevention. A cariogenic oral environment formed by caries without treatment would exacerbate cavities, as has been previously reported [37]. Under these circumstances, it is recommended that parents regularly take their children to the hospital for dental examinations. Additionally, more oral health education, collaboration between parents and schools, and more local medical facilities are oral health strategies in Sichuan Province.

The subjective impression of oral health seems to have good predictive power [38]. A previous study showed significant differences in the “self-assessment of teeth” [28] among 12-year-old students. In the present study, “very poor oral health for self-assessment” was the most strongly weighted variable that contributed to the risk of severe caries. Region, urbanization, and only child were the other three independent predictors of severe caries, which is consistent with previous research [39–41].

An interesting finding was that lifestyle and behavioural factors such as a sugary diet, frequency of tooth brushing, and fluoride toothpaste and dental floss use, which were widely mentioned in dental epidemiological research [42–44], were not included. This difference may be explained differences between the outcome variable in our study (i.e., whether a child had severe caries (DMFT ≥ 3)) and the outcome variables used in previous research [45].

For model validation, we used two CV methods. In general, AUCs lower than 0.6 are considered to have poor discrimination, while AUCs higher than 0.7 suggest high discriminating ability. Both the fivefold and leave-one-out CV methods indicate a good discriminating ability of our nomogram for severe caries.

The strengths of this research are related to its large cross-sectional study design, in which the representative districts and counties of Sichuan Province were selected by a multistage sampling design. In addition, the results of the screening model are displayed with a simple and intuitive graphical form, which facilitates the children and their parents understanding and attracts their attention. However, if the sample size is expanded, the model based on national data will be more practical. Additionally, this model developed herein should be further validated via a longitudinal study.

## Conclusion

Gender, region, urbanization, only child, self-assessment of oral status, and time of last dental visit were shown to be highly correlated with caries risk. The nomogram is a simple-to-use way to identify children who have severe caries (DMFT ≥ 3) and was found to facilitate non-dental professionals in predicting risk values without oral examinations and making referrals to dental professionals.

## Supplementary Information

**Additional file 1**. Overview of the questionnaire

## Data Availability

The dataset of the study is available from the corresponding author at reasonable request.

## Abbreviations

DMFT: Decayed, missing or filled permanent teeth
WHO: World Health Organization
FDI: International dental federation
PPS: Probability proportional to size
CPI: Community Periodontal Index
OR: Odds ratio
CI: Confidence intervals
CV: Cross-validation
FPR: False-positive rate
FNR: False-negative rate
NPV: Negative predictive value
PPV: Positive predictive value
YI: Youden Index
AUC: Area under curve
ROC: Receiver operating characteristic
CRA: Caries risk assessment
